# An Integrated Case Study on Fruit By-Products/Waste Management: Lactic Acid Bacteria Development, Artificial Intelligence Smart Implementation, and Reintegration into Food Chain

**DOI:** 10.3390/foods15152765

**Published:** 2026-08-06

**Authors:** Hiba Selmi, Giovanni Pascoschi, Antonietta Diaferio, Antonio Decataldo, Vittorio Capozzi, Gianluca Follia, Giuseppe Spano, Michele Dassisti, Mariagiovanna Fragasso

**Affiliations:** 1 Department of Agriculture, Food, Natural Science, and Engineering (DAFNE), University of Foggia, 71122 Foggia, Italy; antonietta_diaferio.578411@unifg.it (A.D.); giuseppe.spano@unifg.it (G.S.); 2 BonassisaLab, Z.I. Km. 684.300 Zona Industriale Asi, Incoronata, 71122 Foggia, Italy; 3 Department of Mechanical, Mathematics and Management (DMMM), Polytechnical University of Bari, 70126 Bari, Italy; giovanni.pascoschi@poliba.it (G.P.); a.decataldo2@studenti.poliba.it (A.D.); g.follia@studenti.poliba.it (G.F.); michele.dassisti@poliba.it (M.D.); 4 Institute of Sciences of Food Production, National Research Council (CNR) of Italy, c/o CS-DAT, Via Michele Protano, 71122 Foggia, Italy; vittorio.capozzi@cnr.it

**Keywords:** by-products, *Lactiplantibacillus plantarum*, bio-valorisation, biotechnology, growth prediction, matrix–strain combinations, AI machine learning, cookies, fortification

## Abstract

The increasing generation of agro-industrial by-products from fruit processing represents both an environmental challenge and a valuable opportunity for resource recovery. In this study, tomato, apple, and orange by-products were selected as sustainable substrates for valorization through lactic acid bacteria (LAB) fermentation. The objective was to enhance their functional potential while contributing to waste reduction within a circular bio-economy framework. Selected *Lactiplantibacillus plantarum* strains were applied to each matrix, and microbial growth dynamics were monitored throughout fermentation. In parallel, experimental data were used to build and validate machine learning-assisted models to predict growth kinetics and identify optimal fermentation conditions. To validate the model, the growth of LAB at 32 °C with a bacterial inoculum of approximately 108 CFU/mL was predicted and subsequently compared with experimental results. The developed models demonstrated reliable predictive capability, with low-to-moderate Root Mean Squared Error (RMSE) across different substrates and growth parameters, ranging from 0.143 (vMaxAvg in apple) to 7.155 (LagAvg in orange). Following model validation, selected matrix–strain combinations were applied to develop food products, in which fermented by-products were incorporated as functional ingredients. The resulting formulations exhibited enhanced antioxidant activity and increased total phenolic content, as determined by DPPH (2,2-diphenyl-1-picrylhydrazyl) assay and phenolic analysis, reaching 56.56 mg GAE/100 g (*p* < 0.05) and 33.76 mg Trolox eq./100 g (*p* < 0.05), respectively.

## 1. Introduction

Fruits and vegetables are essential to a healthy diet, providing essential nutrients alongside a wide spectrum of bioactive compounds that contribute to human health. These include vitamins, minerals, dietary fibres, and phytochemicals such as polyphenols, carotenoids, and other secondary metabolites known for their antioxidant and protective properties [1,2]. Among widely consumed plant products, tomato, apple, and orange are particularly important due to their high nutritional value and global production scale, making them key contributors to both dietary intake and agro-industrial processing [3].

In recent years, the continuous growth of the global population, together with the rapid urban development, has led to a rapid rise in waste generation. Thus, the large-scale processing of these commodities generates considerable amounts of waste and by-products in both liquid and solid forms, including peels, seeds, pulp, and pomace. These residues are often discarded or under-exploited despite being rich in valuable compounds [4]. According to the Food and Agriculture Organization of the United Nations, approximately 1.3 billion tons of food are wasted or lost annually. By 2050, this amount is expected to increase significantly, reaching 3.4 billion tons per year [5].

Recently, increasing attention has been directed toward the valorisation of fruit and vegetable by-products within the framework of the circular economy [6]. The circularity concept promotes the reintegration of discarded materials into new production cycles, thereby reducing environmental impact and enhancing resource efficiency [7]. In this regard, numerous studies and methodologies have been developed to valorise fruit by-products for the production of food ingredients, functional foods, pharmaceuticals, and edible films and coatings [8]. Recent examples of valorisation strategies include the use of green processes such as ultrasound, enzyme digestion, and pulsed electric field [9,10]. Methanol extraction coupled with High-Performance Liquid Chromatography with Diode-Array Detection (HLPC-DAD) was effective in extracting Flavanones (202 mmol/kg), flavones (1.39 mmol/kg), and hydroxy cinnamic acids (19.7 mmol/kg) from grape by-products [11]. From citrus, it was possible to recover 6.13% of dry pectin powder yield using ethanol precipitation [12], while from apple pomace, pectin extraction using microwave-assisted extraction yielded 23.32% of dry matter [13]. Ultrasound-assisted enzymatic extraction was efficient in recovering 4.62 mg GAE/g (Gallic Acid Equivalents/gram) of phenolic compounds from apple pomace [14]. Furthermore, microbial fermentation has proven to be a highly effective and sustainable approach. In particular, LAB have been widely applied to enhance the nutritional, functional, and sensory properties of food products. Indeed, their introduction offers a dual advantage: (i) reducing waste, and (ii) generating high-value products [15]. These methods can be used in combination to increase the availability of the compounds of interest. In this context, Baldassa and colleagues explored the influence of microwave pre-treatment on the outcome of 24 h of lactic fermentation of broccoli stalks using the probiotic *Lactiplantibacillus plantarum* CECT 749 [16]. After microwave treatment at 12 W/g for 4 min, a significant increase in total phenolic compounds to 3.4 mg GAE/g dry weight was observed. In general, LAB are widely utilized to enhance the nutritional value and functional properties of various food matrices. Indeed, lactic fermentation acts as a sustainable “clean label” strategy to increase nutrient bioavailability, produce bioactive compounds, and extend the shelf life [17]. However, scaling up the production of bio-based products requires modernizing fermentation technologies [18]. This can be accomplished either by developing microbial strains with enhanced performance or by optimizing key parameters to maximize productivity and overall yields [19]. Together, the increasing complexity of fermentation systems and interactions with the external environment have driven the development and implementation of advanced modelling to better understand and control microbial processes [20]. Indeed, models can be applied to transform process knowledge and data into relevant predicted variables that can be used in real time for monitoring and process optimization [21]. Empirical models, mathematical models based on experimental data and observations, are generally employed to represent the complex biochemistry of cells and interactions between microorganisms and the fermentation environment. Indeed, mathematical and statistical models based on empirical data are commonly employed to describe microbial growth kinetics and substrate interactions in heterogeneous matrices [22,23]. More recently, machine learning and artificial intelligence tools have been introduced to improve predictive accuracy and process optimization. These data-driven approaches are increasingly integrated into decision support systems, enabling the prediction of microbial behaviour, process performance, and product quality under different conditions [24,25].

Within this framework, the present study focuses on the valorisation of fruit by-products, including tomato, apple, and orange matrices, through lactic acid fermentation combined with predictive modelling strategies. Indeed, the main objective is to develop a machine learning-assisted approach capable of monitoring and predicting the dynamics of microbial growth in the selected matrices. By integrating experimental fermentation data with computational modelling, this work aims to identify the most efficient LAB strains for each matrix, thereby supporting the development of a sustainable valorisation process. This combined experimental-computational strategy offers a novel approach to transforming fruit processing residues into high-value resources within a circular economy.

## 2. Materials and Methods

### 2.1. Study Structure

The study was conducted in four phases.

(i) The first phase involved collecting data related to LAB growth in different fruit matrices under various isothermal conditions (25 °C, 30 °C, 35 °C, 37 °C, 45 °C). This phase was conducted at the laboratory scale by inoculating each LAB strain into different fruit by-product juices and monitoring growth under different conditions.
(ii) The second phase employed the analysis of the raw experiment results using the Baranyi–Roberts model and created a predictive model using the Random Forest model. This phase included constructing a structured dataset that effectively analyses raw experimental measurements and develops supervised, unsupervised, and deep learning algorithms for prediction.
(iii) The third phase centred on the exploitation of smart solutions by modelling the growth of LAB strains in different fruit by-products’ juices at a determined isothermal condition (32 °C) with a bacterial inoculum of 0.1 (10^8^ CFU/mL), using both machine learning and experimental laboratory-scale methodologies.
(iv) The fourth phase focused on the use of fruit by-product juices for the development of traditional fermented, traditional non-fermented, and non-traditional functional novel foods. This phase focused on selecting the best strain–matrix combinations and on developing foods by introducing fermented fruit by-products. The food application included the development of plant-based beverages and cookies using barley flour and *Chlorella vulgaris* algae powder.

### 2.2. Lactic Acid Bacteria Strains

LAB strains used for this study are summarized in the table below (Table 1). Ten *Lactiplantibacillus plantarum* (*L. plantarum*), belonging to the Laboratory of Industrial Microbiology collection of the University of Foggia, Italy, were selected based on their functional properties. All strains were previously stored in glycerol 20% (*v*/*v*) at −80 °C. For the experiments, all strains were cultivated in de Man–Rogosa–Sharpe MRS (Oxoid, Basingstoke, UK) and incubated at 37 °C for 24 h under aerobic conditions.

### 2.3. Collection and Treatment of Fruit By-Products-Waste

Defected fruits with unusual size, shape, colour, or blemishes were collected, washed with distilled water, and blended for juice preparation. The obtained juices were further centrifuged to remove solids, and none were sterilized. Fruit matrices include apple, tomato, and orange [26].

Fermentation of fruit juice by-products was carried out at a laboratory scale under sterile conditions to eliminate the influence of external factors and ensure a controlled and reproducible experimental environment. The ten selected strains (Section 2.2) were resuspended in 0.9% (*w*/*v*) sodium chloride solution to obtain concentrations of 10^7^ CFU/mL, 2 × 10^8^ CFU/mL, 10^8^ CFU/mL, or 10^9^ CFU/mL. Each strain, at each inoculum concentration, was then separately inoculated into each fruit juice by-products for fermentation. In total, 30 different fermentations were carried out, excluding the natural fermentations with indigenous microbes. Three replicates were prepared for each fermentation.

### 2.4. Cell Growth in Fruit Juice By-Products: Spectrophotometric Analysis

Microbial growth in fruit juice by-products was monitored by measuring optical density (OD) for 24 h at different temperatures (25 °C, 30 °C, 35 °C, 37 °C, and 45 °C). The absorbance was measured over a wavelength range of 350–650 nm using a SPECTROstar Nano microplate reader (BMG LABTECH, Ortenberg, Germany). OD was measured over a wavelength range to reduce potential interference from juice pigment absorbance with bacterial growth detection. All measurements were collected and used to create the dataset.

### 2.5. Definition of a Data Acquisition System, Database Scheme, and Data Collection

Given the complexity of this structure, a dedicated web platform was developed to upload experiments and store their results in a new, remotely accessible database. The first Excel (Excel 2019, version 2605) files were also imported into this system.

The database contains several tables, among which the most relevant are:

(i) **Experimental_data**, which stores the concentration values for each of the 96 wells, is linked to the experiment via the foreign key filename_ID;
(ii) **Organization_strains_on_plate**, which associates each of the 96 wells with the strain used, again linked by filename_ID;
(iii) **Experimental_conditions**, which provides metadata for each experiment, is uniquely identified by an ID referenced by the other tables.

The main objective of this decision support system is to leverage the experimental data generated by the research group to identify the optimal strain for each matrix in scenarios designed to maximize the recovery of food by-products. The analysis of present experiments was conducted using the Baranyi–Roberts model [27], one of the most widely adopted formulations in predictive microbiology, as it realistically captures the three fundamental phases of microbial dynamics: lag, exponential growth, and stationary phase. The model introduces a physiological state variable that modulates the initial transition, enabling a more accurate estimation of the lag phase than traditional logistic models. In its most common form, microbial growth is expressed as:

$$\ln y\left(t\right)=\ln y_{0}+\;\mu_{max}A\left(t\right)-\ln\left(1+\;\frac{e^{\mu_{max}A\left(t\right)}-1}{e^{\ln K-\ln k_{0}}}\right)$$

in which

$$A\left(t\right)=t+\frac{1}{\mu_{max}}\;ln[e^{-\mu_{max}t}+e^{-h_{0}}-e^{-\left(\mu_{max}+h_{0}\right)}]$$

where y(t) denotes the microbial concentration at time t, y_0_ the initial concentration, K the maximum achievable concentration (plateau), μ_max_ the maximum specific growth rate, and h_0_ a parameter describing the initial physiological state of the inoculum. The lag time λ can be derived as $\lambda =\;\frac{h_{0}}{\mu_{max}}$.

**Figure 1:**
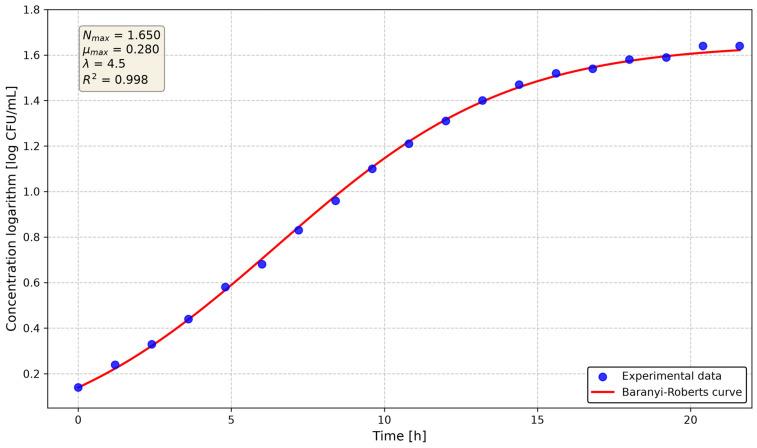
A representative example of an experimental growth curve for orange matrix samples alongside the fitted Baranyi–Roberts model.

Applying the model to time-series data from concentration measurements during food waste recovery allows simultaneous estimation of the three key parameters: the maximum microbial concentration reached, the growth rate during the exponential phase, and the lag time, which quantifies the delay before growth becomes detectable. A representative example is illustrated in Figure 1, which depicts the experimental growth curve for the orange matrix samples alongside the fitted Baranyi–Roberts model. The graph highlights the three characteristic kinetic parameters and their respective confidence intervals.

The ideal scenario would be to achieve the maximum concentration in the shortest possible time, with the highest growth rate. For this reason, all results were stored in a dedicated database, designed to support the application of machine learning and deep learning algorithms to simulate conditions not directly represented in the available experiments.

To facilitate intuitive interaction and integration into more complex solutions, a chatbot interface was conceived, enabling users to query the system in natural language. The deep learning model described in the following section translates these queries into the most suitable database queries. When no matching experiment is found, all data associated with the same matrix are used to train a regression model, which is then used to predict the most plausible outcomes.

The local results database was implemented with a simplified structure, consisting of a single main table, *report*, where each record is identified by a surrogate key (an incrementing integer). The composite key is defined by the matrix identifier (referenced from the *matrici* table, which associates IDs with matrix names), the experiment date, the analyzed strain, and the wavelength used by the spectrophotometer. For each record, the mean values of the three parameters are stored: *MaxAvg* (maximum concentration: the highest bacterial concentration reached during the experiment), *vMaxAvg* (maximum growth rate: the fastest rate at which bacteria multiplied during exponential phase), and *LagAvg* (lag time before the onset of growth: the adaptation period), all estimated by fitting the Baranyi–Roberts model to the well data. The database schema is therefore structured as follows:

- report (**ID**, IDMatrice, Ceppo, Lunghezza d’onda, MaxAvg, vMaxAvg, LagAvg);
- matrici (**ID**, nome).

where the bold attributes denote primary keys.

### 2.6. Development of Supervised, Unsupervised, and Deep Learning Algorithms

The sustainable valorisation of agro-food waste increasingly relies on data-driven methods to support informed decision-making in microbial-based bioprocesses. Accurate selection of microbial strains and the parametrization of experimental conditions require extracting meaningful patterns from time-series growth data generated by multi-well assays. This chapter focuses on the algorithmic toolbox employed to analyze these data: it presents the theoretical foundations of the selected supervised, unsupervised and deep learning techniques and describes how each method was adapted to the specific characteristics of the dataset used in this study. Importantly, this chapter is methodological in scope—it details *what* was applied and *how* it was applied, while quantitative results and their interpretation are reported in a subsequent chapter.

The methodological pipeline adopted here can be summarized in three stages. First, a structured dataset is created by transforming raw time-series measurements into a compact set of summary statistics and experimental metadata. Second, a set of pre-processing operations is applied to render features comparable and to reduce dimensionality while preserving the most informative directions in the data. Third, a combination of clustering algorithms and natural-language-based decision support techniques is introduced to (i) identify groups of similar experimental units (e.g., wells or strains), and (ii) enable interactive queries over the experimental database through local large language models (LLMs). The rationale for this pipeline is twofold: (i) summary statistics capture salient aspects of growth curves with a substantial reduction in dimensionality, and (ii) dimensionality reduction and clustering facilitate exploratory analysis and subsequent automation of strain-selection heuristics.

Before applying machine learning algorithms, it was necessary to construct a structured dataset that could effectively summarize the raw experimental measurements. The original data were collected from multi-well assays in which microbial growth was monitored over time under controlled conditions. Since the raw time-series data contained redundant information and noise, a feature-extraction strategy was adopted to derive compact descriptors that capture the most relevant characteristics of each well. The dataset was designed so that each row corresponds to a well, while each column corresponds to a statistical descriptor or experimental parameter.

The selected features are presented below:

Mean ($\bar{x}$) measures the central tendency of growth values:

$$\bar{x}=\frac{1}{n}\sum_{i=1}^{n}x_{i}$$

Standard deviation (σ) quantifies the variability of values around the mean:

$$\sigma =\sqrt{\frac{1}{n}\sum_{i=1}^{n}{\left(x_{i}-\bar{x}\right)}^{2}}$$

Skewness (S) evaluates the asymmetry of the distribution:

$$S=\frac{1}{n}\sum_{i=1}^{n}{\left(\frac{x_{i}-\bar{x}}{\sigma }\right)}^{3}$$

Kurtosis (K) measures the “peakedness” and tail heaviness:

$$K=\frac{1}{n}\sum_{i=1}^{n}{\left(\frac{x_{i}-\bar{x}}{\sigma }\right)}^{4}$$

Additionally, the median, minimum, and maximum values were included for robustness and boundary characterization. Experimental metadata, such as temperature (T), inoculum rate (I), and maximum growth rate (Vmax), were also incorporated to provide biological interpretability.

Once the dataset was structured, a pre-processing stage was required to prepare the data for machine learning algorithms. This phase ensures that features with different scales and units of measurement can be compared meaningfully, and that redundant correlations among variables are reduced before applying clustering or classification methods. Two complementary techniques were adopted: standard normalization and Principal Component Analysis (PCA). All features were standardized using Z-score normalization:

z=(x−μ)/σ

where *x* is the original feature value, *μ* is the mean of the feature, and *σ* is its standard deviation. This transformation produces a new distribution with mean 0 and standard deviation 1.

After normalization, dimensionality reduction was applied using Principal Component Analysis (PCA), a linear transformation that projects the original correlated variables into a smaller set of uncorrelated variables called *principal components*. Each principal component is a linear combination of the original variables:

$$PC_{j}=w_{j1}x_{1}+w_{j2}x_{2}+\cdots +w_{jp}x_{p}$$

where *PCj* is the j-th principal component, *X*1, *X*2, …, *Xp* are the original standardized features, and *wj*_1_, *wj*_2_, …, *wjp* are the component loadings. The first principal component captures the largest portion of the dataset’s variance.

Once the dataset was standardized and reduced via PCA, the next step was to apply clustering algorithms to identify groups of wells with similar microbial growth behaviours. K-means clustering was selected for its simplicity, computational efficiency, and widespread use in exploratory data analysis. K-means partitions a dataset into K disjoint clusters by minimizing the within-cluster sum of squared distances (WCSS):

$$J=\sum_{k=1}^{K}\sum_{x\in C_{k}}{\left|x-\mu_{k}\right|}^{2}$$

where *Ck* is the set of points assigned to cluster *k*, and *μk* is the centroid of cluster *k*. Clustering was performed in the reduced PCA space to mitigate the curse of dimensionality and improve interpretability.

In addition to PCA, t-distributed Stochastic Neighbour Embedding (t-SNE) was used as a nonlinear dimensionality reduction technique to capture the local structure of the data. t-SNE minimizes the Kullback–Leibler (KL) divergence between the probability distributions of similarities in the high-dimensional and low-dimensional spaces:

$$KL\left(P∥Q\right)=\sum_{i\neq j}P_{ij}\log\left(\frac{P_{ij}}{Q_{ij}}\right)$$

where *Pij* and Qij represent pairwise similarities in the original and reduced spaces, respectively, after computing the low-dimensional representation with t-SNE. K-means clustering was then applied to identify cluster structures in this simplified representation.

The design and implementation of the decision support system (DSS) reached maturity through the definition of an integrated architecture that combines natural language processing, database management, and machine learning prediction into a cohesive platform aimed at assisting domain experts in the selection of bacterial strains and experimental conditions to maximize the valorisation of food waste.

After extensive exploratory trials with supervised and unsupervised algorithms such as Principal Component Analysis, t-SNE, and clustering, as well as preliminary experiments with both cloud-based and LLMs, the solution evolved toward a hybrid system centred on the adoption of the Qwen Coder 2.5 model. This model was selected because of its ability to bridge the gap between human operators and complex experimental data, transforming natural language queries into executable SQL statements while maintaining precision, reliability, and interpretability. Unlike general-purpose LLMs that are broadly trained on diverse text corpora, Qwen Coder 2.5 was specifically optimized on large-scale repositories of code and programming-related documentation, such as GitHub v.3.21 and Stack Overflow (https://stackoverflow.com/questions, accessed on 26 July 2026), endowing it with an exceptional capacity to generate context-aware, syntactically correct, and semantically meaningful code fragments.

Its Transformer-based multi-layer self-attention architecture allows it to capture long-range dependencies across structured information, thereby enabling the model not only to understand the relational schema of the database but also to produce SQL queries that combine data from multiple tables, apply grouping, and execute conditional logic in line with domain-specific requirements. One of the key achievements of this approach is its ability to make experimental data accessible to non-technical users. Through the DSS interface, experts unfamiliar with database query languages can formulate their questions in natural language, such as asking which strain performed best on a given matrix under certain conditions, and the system can generate optimized queries to retrieve and present the relevant information.

At the same time, the model proved robust enough to handle complex prompts requiring the integration of multiple pieces of information, passing benchmarks that had previously exposed the limitations of alternative solutions.

The system’s backend was developed using Flask, a lightweight yet flexible Python framework that facilitated integration across the different technological layers. It established a secure, thread-safe connection to the SQLite database, chosen for its portability and simplicity, while also enabling orchestration of communication with the Ollama runtime, which hosts the local instance of the Qwen Coder 2.5 model (Figure S1).

The backend logic included mechanisms to dynamically scan the database schema at runtime, thereby providing the model with explicit information about the data structure, including table names, column types, and implicit relationships. This ensured that query generation was not only syntactically correct but also semantically grounded in the actual dataset. In addition to user-driven questions, the backend implemented a suite of predefined queries that address frequently encountered analytical tasks, such as counting experiments, listing available matrices, and ranking strains by performance metrics. These predefined routines improved usability by providing immediate access to key insights without manual input, while still allowing advanced users to pose custom natural language questions for more exploratory analysis.

An additional component of the backend was the predictive modelling pipeline, which extended the functionality of the DSS beyond simple data retrieval.

Recognizing that not all possible experimental conditions had been tested, and that users would often need to infer results under novel scenarios, the system incorporated machine learning regressors trained on historical experimental data. Random Forest models were chosen for their ability to capture nonlinear relationships and handle heterogeneous data distributions. To predict microbial kinetic parameters, a Random Forest regression model was developed using a structured dataset comprising AAA data points. To ensure robust model evaluation, the dataset was partitioned into a training set (80%) and a testing set (20%). Model tuning and validation were performed employing a 5-fold cross-validation strategy on the training subset. Hyperparameter optimization was conducted via RandomizedSearchCV, focusing on optimizing key parameters, including the number of estimators (ranging from AAA to AAA), the maximum tree depth (set to AAA), and the minimum samples required to split an internal node (set to AAA). Furthermore, to mitigate the risk of overfitting and ensure model generalizability, early stopping techniques were implemented. The models were trained to predict three critical kinetic parameters—maximum concentration, maximum growth rate, and lag time—using temperature and inoculation rate as input variables. The results are presented alongside confidence intervals derived from the diversity and density of the historical experimental data, enabling users to contextualize the predictions based on data robustness accurately. A representative predictive output for the apple matrix is shown in Figure S2.

This predictive functionality was designed to operate in two complementary modes. In the forward inference mode, users specified experimental conditions and received predictions of growth performance, whereas in the reverse inference mode, the system suggested optimal conditions for temperature and inoculation rate for a given strain–matrix combination, effectively guiding experimental planning. In both cases, outputs were carefully structured to maximize interpretability: results included colour-coded confidence indicators, explicit notes when data scarcity limited reliability, and quick insights summarizing the top-performing strains or conditions.

The front-end was designed with a strong emphasis on usability, accessibility, and visual clarity. Built with HTML5, CSS3, and vanilla JavaScript enhanced with Bootstrap 5.3, the interface provided a modern, responsive, and professional environment for interaction. A dual-column layout was implemented, with the left panel containing quick-access predefined queries, predictive modelling tools, and a dynamically generated database schema, and the right panel dedicated to interactive querying and result visualization. The design incorporated dynamic theming, allowing seamless switching between light and dark modes, with preferences stored locally to persist across sessions. Query results were rendered as responsive tables with sorting and filtering capabilities, expandable experiment details, and visual indicators of confidence levels. The system also included modal editors that allowed advanced users to refine auto-generated SQL queries before execution, balancing automation with human oversight. The user experience was further enriched by asynchronous communication with the backend via modern fetch APIs, ensuring smooth interaction without page reloads and providing immediate visual feedback through loading indicators and contextual alerts. Importantly, results presentation was tailored to the nature of the query: structured datasets were displayed as sortable tables, explanatory notes and confidence metrics accompanied predictions, and quick insights highlighted the best-performing options in relation to specific goals.

From a methodological perspective, integrating Qwen Coder 2.5 with the Flask–SQLite–Bootstrap pipeline represented a significant step forward in the development of AI-assisted decision support systems for microbial biotechnology. The chosen architecture addressed several limitations identified in earlier phases. While unsupervised clustering methods struggled to provide interpretable insights and general-purpose LLMs returned inconsistent or unreliable outputs, adopting a specialized coder model ensured both accuracy and reliability in query generation. At the same time, the inclusion of a predictive machine learning component ensured that the DSS could generate value even in data-scarce scenarios, extending its utility beyond retrospective analysis into proactive experimental design. Importantly, the system was deployed entirely on local infrastructure, eliminating privacy concerns associated with sending sensitive experimental data to external servers and ensuring compliance with data governance requirements. The modularity of the architecture also leaves ample room for future improvements. For example, as more experimental data become available, the Random Forest regressors could be retrained with broader coverage, potentially complemented by neural models capable of capturing more subtle interactions. Similarly, while fine-tuning of the Qwen Coder 2.5 model was not feasible within the constraints of the current dataset, the availability of larger proprietary corpora in the future could enable domain-specific adaptations, improving the system’s ability to generate context-sensitive queries and explanations. The architecture could also be extended to incorporate multi-objective optimization, enabling the DSS to suggest strains and conditions that simultaneously maximize multiple performance indicators, such as growth rate and yield, while minimizing lag time.

Taken together, the final solution embodies a novel synthesis of natural language interfaces, structured database management, and predictive analytics. By lowering barriers to accessing experimental data, empowering users with predictive capabilities, and providing a secure, interpretable, and extensible environment, the system demonstrates the potential of AI-driven decision support to transform microbial biotechnology applications in the food industry. The combination of Qwen Coder 2.5 for natural language query generation, Flask for backend orchestration, SQLite for lightweight but robust data management, Random Forest regressors for predictive modelling, and a responsive web-based front-end for user interaction resulted in a system that is both technically advanced and practically usable. This integrated platform not only supports day-to-day decision-making but also provides a scalable foundation for future research and industrial deployment, aligning with the broader objectives of sustainable resource utilization and intelligent automation in the agri-food sector.

### 2.7. Exploitation of Smart Solutions to Support Decision-Making in Selected Fruit-Related Case Studies

To validate the developed predictive model, the DSS platform was asked to predict the growth curves of all strains under specific conditions (32 °C with a bacterial inoculum of 0.1 for 24 h). Together, in a laboratory-scale experiment, the fermentation of fruit juice by-products using 10 different LAB strains was monitored at 32 °C, and growth curves were measured using optical density (OD) in each well over a wavelength range, from 350 nm to 650 nm, with a microplate reader.

Bacterial counts for LAB strains in fruit juices were recorded after 1 day of fermentation. For microbial enumeration, fermented juices were combined with 0.85% sodium chloride solution at a 1:9 (*w*/*v*) ratio and vortexed for 2 min [28]. After that, serial decimal dilutions of the homogenate were prepared, inoculated in MRS plates (in triplicate), and incubated for 24 h at 37 °C. For the control, non-inoculated juices were used, as mentioned above.

### 2.8. Re-Use of Fremented Fruit Juice By-Products in Selected Food Matrices

In this section, three categories of food type were produced: (1) traditional fermented food, (2) traditional non-fermented food, and (3) non-traditional functional novel food. One example from each category was processed at the laboratory scale; traditional fermented foods include a barley-based beverage, traditional non-fermented foods include cookies, and non-traditional functional novel foods include cookies formulated with *Chlorella vulgaris* algae powder.

LAB strains showing the best growth in fruit juice by-products are summarized in the table below (Table 2), and strain–matrix combinations were used to produce a fortified plant-based beverage. Barley flour (Bongiovanni S.r.l., Villanova Mondovì, Italy) and algae *Chlorella vulgaris* powder (Sevenhills Wholefoods, Wakefield, UK) were purchased online and stored in a dry, ambient environment.

(1) Following the method outlined by Cirat and colleagues [29], *Chlorella vulgaris* algae powder was added to the barley flour in a proportion of 1.5% (*w*/*w*), and sterile water was added at a ratio of 1:4 *w*/*v* (kg/L), and the beverage was further incubated 30 min at 60 °C in a water bath for gelatinisation. After cooling to room temperature, the barley beverage was divided into 200 mL portions and transferred to sterilized glass bottles. In each bottle, fermented fruit juice containing LAB strain-fruit by-product was added at a concentration of 1% (*v*/*v*). Regarding microbial load, *Lpb. plantarum* strains were added at a concentration of 10^7^ CFU/mL. Bottles were kept at 37 °C for approximately 7 h until the pH reached <4, and then stored at 4 °C for 1 week.
(2) Regarding cookie formulation, we followed the method outlined by Pahane and colleagues, modified [30]; cookies were produced according to the formulation presented in the table below (Table 3). All dry ingredients were mixed in a stainless-steel pan. The margarine was later added, and the mixture was blended manually until the dough homogenized; water and 48 h fermented fruit juices’ by-products containing 10^7^ CFU/mL of *Lpb. plantarum* strains were added. The dough was cut and weighed (7 g/biscuit). Biscuits were baked on a lightly greased aluminum baking tray in a 220 °C, ventilated, multifunction gas oven for 15 min. The samples were removed from the oven and cooled to room temperature on a cooling rack. The analyses were carried out on fresh biscuits.
(3) Following the method outlined previously for cookie production and ingredients described in Table 3, 3% of *Chlorella vulgaris* was added to the dough.

For the microbiology analysis, several media of growth were used: MRS agar, Sorbitol McConkey Agar (SMAC), Plate Count Agar (PCA), and Potato Dextrose Agar (PDA), purchased from Oxoid, Basingstoke, UK, and Polymyxin Acriflavine Aithium Chloride ceftazidime Aesculin Mannitol agar (PALCAM) purchased from Merck KGaA, Darmstadt, Germany. PCA is a non-selective medium used to quantify total viable bacteria, whereas PDA is a selective medium for the enumeration of yeasts and moulds. MRS agar, PALCAM, and SMAC are selective media used for the isolation and quantification of LAB, *Listeria* spp., and pathogenic *Escherichia coli*, respectively. For MRS agar, plates were incubated at 37 °C for 48 h; SMAC plates were incubated in aerobic conditions at 37 °C for 24 h; PCA plates were incubated at 30 °C for 48 h, while PDA plates were incubated at room temperature (about 25 °C) for 4 days. Regarding PALCAM plates, dilutions were incubated at 37 °C for 48 h. Controls inoculated with a single bacterial culture in the corresponding medium were also analyzed. Briefly, serial dilutions were made with 0.85% physiological solution, reaching 10^−10^. Dilutions were inoculated in spot-on agar media (20 µL/spot) and incubated at the corresponding parameters (temperature and duration) of each microbiological growth medium.

The analysis of the antioxidant activity and the total phenolic content of food products were carried out on samples extracted as follows: 5 g of food sample was homogenized in 20 mL methanol/water solution (80:20 *v*/*v*) for 2 min, using a homogenizer (T-25 digital ULTRA-TURRAX^®^-IKA, Staufen, Germany) and then centrifuged (Prism C2500-R, Labnet, Edison, NJ, USA) at 15,000 rpm for 5 min at 4 °C. The extracts were collected and stored at −20 °C before the analysis.

The antioxidant activity was evaluated using the DPPH radical (1,1-diphenyl-2-picrylhydrazyl) scavenging assay, following the method described by Cozzolino et al. [31]. After appropriate dilution, the supernatant was mixed with a DPPH solution (0.4 mmol/L) to initiate the reaction, and the absorbance was measured after approximately 30 min at 515 nm. Trolox was used as a standard, and the antioxidant activity was reported in mg of Trolox equivalents per 100 g of fresh weight (mg Trolox eq./100 g).

Total phenolic compound content was determined using the Folin–Ciocalteu colorimetric assay according to the protocol of Cozzolino and colleagues [31]. The food extract was mixed with deionized water (*v*/*v*) and the Folin–Ciocalteu reagent (*v*/*v*). The mixture was incubated in the dark for 2 h, and the absorbance was measured at 765 nm. Total phenolic content was assessed based on the gallic acid standard curve and expressed as mg GAE per 100 g.

### 2.9. Data Analysis

#### 2.9.1. Model Performance Analysis

The performance of the DSS and the reliability of predictive models were evaluated using standard statistical metrics. The architecture was implemented in Python 3.12, with Flask for backend orchestration and HTML5/CSS3 for the user interface. Natural language interaction was enabled through the locally deployed Qwen Coder 2.5 via Ollama, ensuring data privacy and compliance. Model performance was assessed using the coefficient of determination (R^2^) applied to the Baranyi–Roberts model to evaluate goodness-of-fit. This metric is defined as follows:

$$R^{2}=1-\frac{\sum_{i=1}^{n}{\left(y_{i}-\hat{{\hat{y}}_{i}}\right)}^{2}}{\sum_{i=1}^{n}{\left(y_{i}-\bar{y}\right)}^{2}}$$

where y_i_ denotes the observed concentration logarithm, ${\hat{y}}_{i}$ represents the value predicted by the kinetic model, and $\bar{y}$ is the mean of the experimental observations. To evaluate the accuracy of the Random Forest regression models in predicting continuous kinetic variables—specifically the maximum concentration (MaxAvg), maximum specific growth rate (vMaxAvg), and lag time (LagAvg)—the Root Mean Squared Error (RMSE) was employed as the primary performance indicator.

$$RMSE=\sqrt{\frac{1}{n}\sum_{i=1}^{n}{\left(y_{pred,\;i}-y_{obs,\;i}\right)}^{2}}$$

The RMSE was used to quantify discrepancies between predicted and experimental values. In addition, a confidence metric was implemented to assess prediction robustness under varying conditions, reflecting the quality and density of the training data. Reliable predictions depend on high-quality model fitting (high R^2^), as excessive data noise limits the identification of meaningful biological patterns.

#### 2.9.2. Statistical Analysis

Statistical analysis for the experimental section (Section 2.4) was performed using GraphPad Prism software 6.0 (GraphPad Software Inc., San Diego, CA, USA). Analysis of variance one-way ANOVA was used to compare data with a single factor across groups, and two-way ANOVA was used to compare multiple factors across groups Differences were adjudicated using the post hoc analysis recommended by GraphPad Prism 6.0. All experiments were performed in duplicate or triplicate and carried out in a randomized design.

## 3. Results and Discussion

### 3.1. Release of the Experimental Dataset: LAB Strains Versus Fruit By-Products

Initially, we analyzed the stored database data, which were derived from the Baranyi–Roberts modelling of the acquired experimental data. The orange matrix was selected for analysis as it represents the substrate upon which the greatest number of systematic experiments were conducted (Table 4).

The dataset used in this study was constructed from controlled laboratory experiments designed to investigate the effect of temperature and inoculum ratio on microbial growth during the valorisation of food waste. These two variables were selected as input features because they are consistently reported as the most influential operational parameters governing microbial kinetics, metabolic activity, and process stability in food waste fermentation systems. Temperature directly affects enzymatic activity and cellular metabolism, while the inoculum ratio determines initial microbial density, competition dynamics, and adaptation time, all of which strongly influence maximum biomass concentration, growth rate, and lag phase duration [1]. Table 5, Table 6 and Table 7, presented below, illustrate the database entries according to the methodology described in the previous chapter. Each table caption also includes the mean R^2^ value as an indicator of the goodness-of-fit between the theoretical model and the experimental data. In some instances, data are absent as the corresponding experiment was not performed.

In the following section, we used the previously constructed ML model to predict the growth parameters of the *Lpb. plantarum* strain in apple, tomato, and orange by-products. Parameters include MaxAvg, vMaxAvg, and LagAvg. According to the prediction data presented in Table 8, Table 9 and Table 10, the strain SF2A35B showed the highest MaxAvg (MaxAvg = 1.343), followed by the strain ATCC8014, while the strain B01 exhibited the lowest MaxAvg rate with a value of 0.194 at 32 °C with a bacterial inoculum of 0.1 (10^8^ CFU/mL) in the apple matrix. Similarly, in tomato by-product juice, MaxAvg values ranged from 1.699 to 1.204, with strains ATCC8014 and SD47 exhibiting the highest and lowest MaxAvg rates, respectively. The strain ATCC8014 showed a high MaxAvg on the orange matrix, followed by SD47 with values of 0.856 and 0.839, respectively. As expected, strains showing MaxAvg values exhibited the maximum growth rate (vMaxAvg), except strain MM1, which showed the highest vMaxAvg on the tomato matrix. In the orange matrix, it is predicted that MM1 exhibited the highest growth rate, reaching 0.428, while experimental findings revealed that MM1 vMaxAvg reached 0.62, which was not the highest value; the strain M5MA1 exhibited the highest vMaxAvg on orange juices (Table 11). The confidence level in all fruit matrices ranged from 31.4% to 0.89% (Table 12), in which all strains showed a higher confidence level in the apple matrix than in the tomato and orange. The limited number of experiments might explain the variation in the prediction’s confidence level.

The selection of optimal strains for further experiments was based on the strain profile (previous publications and laboratory findings), the prediction and experimental results, mainly the confidence level, and the coherence of findings. Parameters, including the highest MaxAvg (greatest final biomass), the best vMaxAvg (fastest growth rate), and the shortest LagAvg (fastest adaptation to the fruit juice matrix), were applied as well.

For apple by-products, the strain SD47 showed the highest confidence level (89.4%), followed by the strain SF2A35B (87.2%), while the strains ATCC8014 and WCFS-1 showed a modest confidence level in prediction. The strain SD47 expressed a high MaxAvg of about 0.605, which was confirmed through experimental findings, with a low LagAvg (11.456 and 12.952 in predicted and experimental data), leading to its selection for apple by-product fermentation. As explained previously, LagAvg is the estimated time from the adaptation phase to the onset of growth. Table 11 presents measured and predicted viable LAB counts for all fermentation combinations, reflecting AvgMax, vMaxAvg, and LagAvg values. All strains showed the ability to grow in all matrices, with varying potentials.

In tomato by-products, the confidence level ranged from 36.7% to 66.5%, with the strain WCFS-1 exhibiting the highest confidence level, followed by the strain V0102121A9. However, WCFS-1 showed a modest MaxAvg value of about 1.371, while the strain SF2A35B showed interesting MaxAvg and LagAvg values of 1.675 and 6.95, respectively. These findings were later affirmed by the experimental findings, which were 0.975 and 8.26 for MaxAvg and LagAvg, respectively.

Zooming in on orange by-products, the confidence level, MaxAvg, and vMaxAvg ranged between 31.4 and 43.9%, 0.46–0.919, and 0.27–0.43, respectively (Table 8, Table 9 and Table 12). Although the strain M5MA1, with the highest confidence level, exhibited the lowest LagAvg value, it requires more time to adapt. While strain MM1 exhibited the highest AvgMax value, it had a modest confidence level of about 31.9%, as confirmed by experimental findings. The strain SD47 exhibited a prediction confidence level of 43.7%, with an interesting predicted MaxAvg value of about 0.84, which was confirmed by experimental findings. Regarding vMaxAvg, the strain SD47 exhibited the fastest growth rate. In experimental findings, the strain CECT8944 showed the lowest LagAvg with a value of 8.725, but it exhibited a relatively low confidence level. Therefore, the best candidate for the orange matrix was the strain SD47.

According to Table 13, the RMSE values indicate generally good agreement between experimental and predicted data, although model performance varied depending on the substrate and growth parameter considered. The lowest prediction errors were observed for apple by-products, with RMSE values of 0.249 for MaxAvg, 0.143 for vMaxAvg, and 2.995 for LagAvg, highlighting the model’s high accuracy, particularly in predicting the maximum growth rate. For tomato substrates, moderate prediction errors were observed, with RMSE values of 0.806 (MaxAvg), 0.377 (vMaxAvg), and 3.066 (LagAvg), suggesting acceptable but less-precise model performance. Higher variability was observed for orange by-products, especially for the lag phase, where the RMSE reached 7.155, compared to 0.304 for MaxAvg and 0.450 for vMaxAvg. This increase in prediction error for LagAvg may be attributed to inherent biological variability and to the microbial adaptation phase’s sensitivity to environmental conditions. Overall, these findings demonstrate that while the predictive model is robust, its accuracy is influenced by the specific characteristics of each by-product matrix.

### 3.2. Development and Evaluation of Supervised, Unsupervised, and Deep Learning Algorithms

The application of supervised learning algorithms was severely limited. Specifically, a classification approach is not applicable for selecting the strain that maximizes growth on a given matrix, as this objective requires the prediction of a continuous variable, the MaxAvg parameter (defined as the mean of the maximum growths observed in the wells treated with the same strain). However, the construction of a sufficiently accurate regression model is hindered by the scarcity of available descriptive variables. The only features potentially usable for model training were the temperature, the inoculum rate, and the condition of sterility.

Given this limitation, a regression approach was attempted, despite the low dimensionality of the feature space, using a Random Forest model. This model allowed prediction of the MaxAvg parameter even for combinations of the three parameters not present in the training dataset, including the specific matrix and strain under examination as inputs. Although the regressor thus constructed represents a first attempt at modelling the problem, the reliability of its predictions remained limited by the scarcity of available information. For this reason, while its exploratory potential was recognized, this approach was not adopted as the definitive solution within the project.

Preliminary exploratory data analyses were initially conducted using a comprehensive suite of unsupervised machine learning algorithms, including Principal Component Analysis (PCA), K-means clustering, t-distributed Stochastic Neighbour Embedding (t-SNE), Density-Based Spatial Clustering of Applications with Noise (DBSCAN), and Agglomerative Hierarchical Clustering. As extensively detailed in our previous foundational study focusing on pre-factorial data, these nonlinear dimensionality reduction and clustering techniques struggled to delineate interpretable, robust clusters for complex strain–matrix interactions in an autonomous manner. The inherent biological complexity and multidimensional variance of microbial growth dynamics rendered purely unsupervised heuristics insufficiently interpretable for precision strain selection.

Consequently, to maximize the valorisation of the novel factorial dataset presented in this study, the methodological focus was strategically shifted. The current framework relies entirely on the hybrid deep learning and supervised predictive architecture (Qwen Coder 2.5 and Random Forest regressors) described in Section 2.6. This transition successfully overcomes the limitations of exploratory clustering, providing actionable, high-confidence decision support that effectively translates raw experimental kinetics into optimized industrial parameters.

Compared to traditional predictive microbiology approaches, such as Response Surface Methodology (RSM) or classical secondary kinetic modelling, the proposed AI-assisted framework presents distinct advantages for agro-industrial by-product valorization. While RSM relies on rigid experimental setups (e.g., Central Composite or Box–Behnken designs) and is highly effective for static optimization, it often struggles to accommodate the highly nonlinear biological variance across multiple heterogeneous matrices [32,33]. Conversely, the Random Forest algorithm natively captures complex, nonlinear interactions between operational parameters (temperature, inoculum rate) and microbial performance across different strains [34]. Furthermore, whereas traditional secondary kinetic modelling requires continuous manual parameter adjustments when new strains or substrates are introduced, the implemented machine learning architecture dynamically integrates new experimental data, iteratively refining its predictions. This continuous learning capability makes the AI-driven decision support system particularly suitable for circular bio-economy processes, where raw material variability remains a primary challenge.

### 3.3. Exploitation of Smart Solutions to Support Decision-Making in Selected Fruit-RelatedCase Studies

This decision support system is designed to identify the optimal bacterial strain to enhance recovery of a given food matrix under specific temperature and inoculum rate conditions.

The first case study aimed to determine: (i) the strain with the shortest lag time, (ii) the strain with the highest growth rate, and (iii) the strain with the largest overall growth, as well as to identify the best-performing strain, considering all three parameters simultaneously. Furthermore, the study investigated whether it was possible to reduce the inoculum rate while maintaining the strain’s performance.

**Figure 2:**
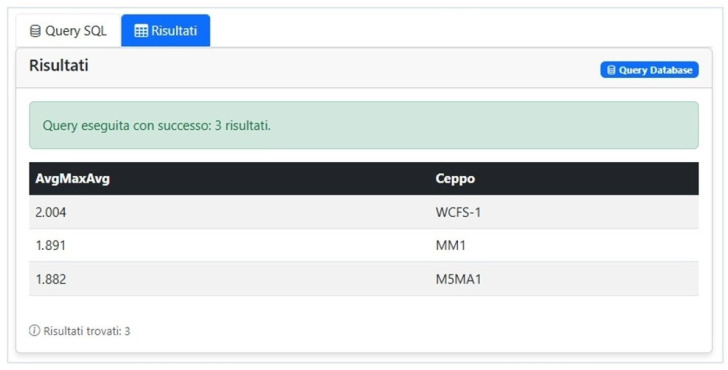
Results obtained on the user interface. SQL query results table showing the top bacterial strains (*Ceppo* → *Strain*) ranked by the AvgMaxAvg metric. The interface confirms successful query execution (*Query eseguita con successo* → *Query executed successfully*) and displays the retrieved results (*Risultati* → *Results*).

The selected matrix was orange because it provided the most experimental data points, ensuring more reliable results. Since no temperature information was specified, ambient temperature (25 °C) was adopted (Figure 2).

**Figure 3:**
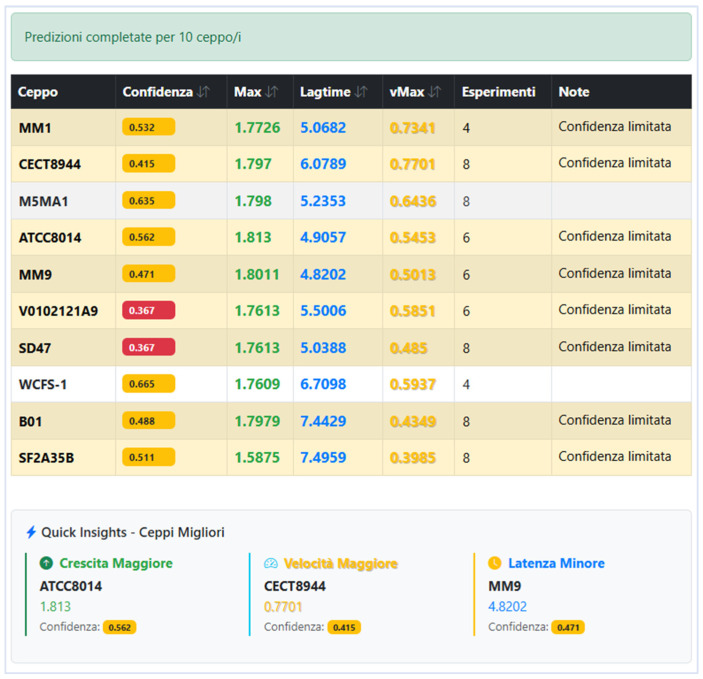
Results obtained for all food matrices on the user interface. Predicted growth model parameters for 10 strains. The table summarizes the predicted values for each strain (*Ceppo*), including confidence (*Confidenza*), maximum growth (*Max*), lag time (*Lagtime*), maximum growth rate (*vMax*), number of experiments (*Esperimenti*), and notes (*Note*). Strains with lower confidence values are flagged as limited confidence (*Confidenza limitata*). The Quick Insights – Best Strains (*Ceppi Migliori*) panel highlights the strain with the highest growth (*Crescita Maggiore*; ATCC8014, Max = 1.813), the highest growth rate (*Velocità Maggiore*; CECT8944, vMax = 0.7701), and the shortest latency (*Latenza Minore*; MM9, Lagtime = 4.8202).

Furthermore, the proposed decision support system can be utilized to predict outcomes under specific experimental conditions by generating targeted database queries. In instances where the required parameters are not present in the existing dataset, the system leverages its machine learning component to simulate unexplored scenarios, such as the behaviour of previously untested strains. For example, by configuring the model with a temperature of 35 °C and an inoculum rate of 0.5 (0.5 × 10^8^ CFU/mL), the predictive results illustrated in Figure 3 are obtained.

The “Quick Insights” flashcards at the bottom of the interface highlight the top-performing strains for each of the three kinetic parameters, though the main table can be sorted by any of these variables. Interestingly, while MM1 emerged as a strong overall performer across all three parameters, it did not rank as the absolute best in any single category. This distinction allows the end-user to prioritize specific industrial or experimental goals: for instance, selecting ATCC8014 to maximize total biomass yield, or MM9 to minimize process duration.

However, it should be noted that the prediction confidence for MM1 remains limited (0.532), as it is based on only four recorded experiments. Despite this, the strain exhibited a notably high growth rate and an exceptionally short lag phase compared to other strains tested on the same matrix, even while showing relatively low overall growth.

**Figure 4:**
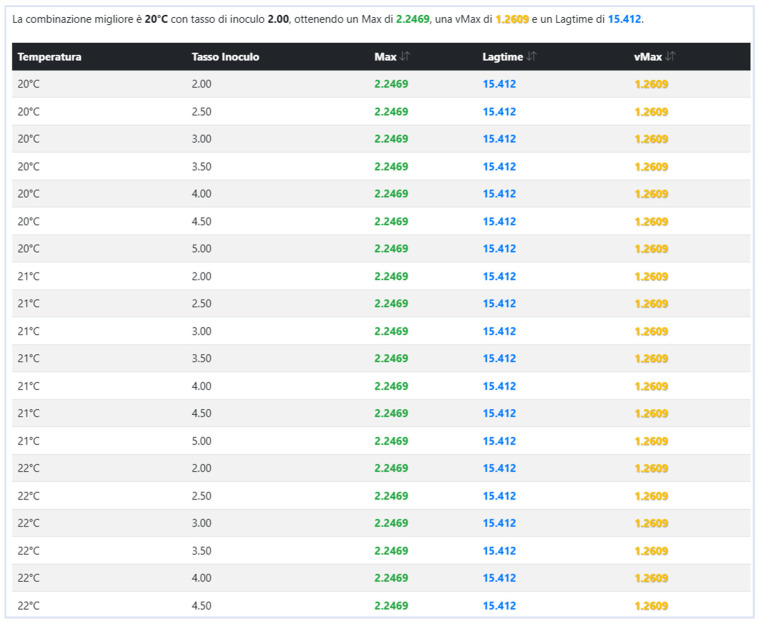
Results of the strain showing the highest confidence level.

To demonstrate the DSS’s backward inference capabilities, the subsequent analysis focused on strain M5MA1, which exhibits a more robust profile with a higher confidence level (0.635) and a larger experimental dataset (n = 8). Using the parameter selection interface, a comprehensive simulation was performed, covering temperatures from 20 °C to 35 °C (1 °C increments) and inoculum rates from 0.5 to 10 (0.5 increments) (equivalent to 0.5 × 10^8^ to 10^9^ CFU/mL). The results of this sensitivity analysis are reported in Figure 4. The simulation identified an optimal configuration at 20 °C with an inoculum rate of 2.0 (2 × 10^8^ CFU/mL), conditions that yield significant improvements in both lag time and growth rate (Figure 4).

The three tables collectively summarize the machine-learning-predicted growth behavior of the ten *Lactiplantibacillus plantarum* strains using tomato by-product as a model matrix under different combinations of temperature and inoculum rate. Together, they provide a broad overview of three complementary kinetic descriptors: maximum growth capacity, maximum growth rate, and lag phase duration. Table 14 reports the predicted MaxAvg values, representing the expected maximum growth reached by each strain under the tested tomato fermentation conditions. Overall, the values indicate that the tomato matrix can support growth of all strains, although the predicted maximum biomass varies according to both strain identity and process conditions. Table 15 presents the predicted vMaxAvg values, which describe the maximum growth rate of each strain. These data show that the fastest predicted growth does not necessarily coincide with the highest predicted final biomass. However, evaluating process speed is important for process economics and the dominance of the inoculated strain. Table 16 reports the predicted LagAvg values, corresponding to the estimated adaptation time before active growth begins. Lower LagAvg values indicate faster adaptation to the tomato matrix. Some conditions favor shorter adaptation times across multiple strains, while others result in longer predicted lag phases. Taken together, the three tables provide a multidimensional prediction profile for LAB growth in tomato by-products. Table 14 helps identify conditions favoring high final biomass, Table 15 highlights conditions associated with rapid growth kinetics, and Table 16 clarifies the expected adaptation behavior of the strains. Therefore, the combined interpretation of these tables supports a multicriteria approach to strain selection (Table 14, Table 15 and Table 16).

### 3.4. Re-Use in Selected Food Matrices

This section introduces fruit juice by-products as functional ingredients for the formulation of novel functional foods. After lactic fermentation of fruit matrices, including apple, tomato, and orange, the fermented juice was used in food formulation. The food products include traditional fermented foods, traditional non-fermented foods, and non-traditional functional foods.

Starting with a fermented barley-based beverage, the ability of the selected LAB strains to survive, grow, and produce organic acids in barley beverages was investigated. During the fermentation process, pH levels were measured at T0 (before fermentation) and T1 (after 8 h of fermentation). Results are summarized in the table below (Table 17). A decrease in pH was observed in all beverages, reaching pH 4 after 8 h of incubation, which confirms the potential of the selected combination to ferment barley flour. Indeed, the pH variation generally reflects the metabolic activity of microorganisms during fermentation. After about 8 h, all combinations showed acidification, reaching a final pH of about 3.75, while the strain *Lpb. plantarum* SD47 exhibited a pH of about 4.0. This variation highlighted chemical interactions and transformations of primary compounds and molecules with the generation of novel products [35], while bacterial counts were increasing, which can indirectly reflect these transformations.

In this regard, all strains exhibited a similar growth pattern, and the LAB count in the fermented barley-based beverage ranged from 10.71 to 11.71 log CFU/mL (Table 18). Findings revealed an increase in LAB strains count compared to the initial inoculated concentration (about 7 log CFU/mL), and no harmful microbes were detected on the selective medium in fermented barley beverage (Table 18). Indeed, total bacterial count in fermented barley beverage ranged between 3.15 ± 2.37 and 7.85 ± 5.94 log CFU/mL, while moulds and yeasts count ranged between 0.0 and 0.8 ± 0.09 log CFU/mL. Zooming in on the bacterial count on MRS agar plates, the number of colonies was significantly higher than the number of microbes on PCA and PDA plates. This can be explained by either the highly nutrient-rich content of the MRS medium which is rich in simple sugars (glucose/dextrose), peptones, and yeast extract, making it an excellent environment for fungi to thrive, as well as by the absence of any other microbe except the inoculated LAB strains. This is affirmed by the very low count of moulds and yeasts in barley beverage fermented by LAB strains. Regarding both controls, barley non-inoculated and barley non-inoculated, fortified by algae, the level of moulds and yeasts was 6.35 ± 2.48 log CFU/mL and 4.35 ± 3.89 log CFU/mL. These results proved the potential of our *Lpb. plantarum* strains to control/limit the growth of moulds and yeasts through the creation of stressful conditions by reducing the pH. Regarding cookies prepared with barley and fermented fruit juice by-products (cookies fortified and not-fortified with *Chlorella vulgaris* powder), matrices were analyzed after baking: microbiological analysis revealed a significant reduction in LAB strains’ viability from 7 log CFU/mL (before baking), reaching 0.95 ± 0.52–2.42 ± 1.76 log CFU/g in baked cookies, detected on MRS agar plates. Regarding PCA and PDA agar plates, colonies were present in lower levels/numbers, and no significant difference was detected in colony number among PCA, PDA, and MRS plates, proving colonies can be LAB strains. Similar findings revealed a decrease in LAB viability from 9.45 to 3.53 log CFU/g after baking [36]. Zhang and colleagues found a 4 log CFU/g decrease in probiotic viability after baking, whilst, during storage, LAB strains reactivated and the probiotic count increased [37]. No pathogens were characterized. The number of LAB strains were decreased compared to the initial inoculum concentration before baking. The high baking temperature might explain the decrease in LAB count in cookies prepared with *Chlorella vulgaris*, which was higher than in those prepared without algae fortification. Findings reported that adding *Chlorella vulgaris* at a concentration of 1.5% (*w*/*v*) to LAB media during growth accelerated logarithmic growth and influenced the enzymatic activity of the strain [38]. Further studies showed that LAB strains grow better in the presence of Chlorella powder, with higher protein concentrations, and that this increases their survival rates under stressful conditions [39]. In a barley-based beverage, fermented apple juice by-product exhibited the highest LAB growth with a value of 10.71 ± 9.74 log CFU/mL. In this regard, Rizzi and colleagues found that the concentration of the strain *Lpb. plantarum* KABP051 in apple juice was better than in orange and peach juices. These findings are in line with several findings highlighting the potential of LAB to ferment apple juices [40]. It was reported that *Lpb. plantarum* can improve the antioxidant capacities of the juice by producing lactic acid and metabolizing phenolic compounds [41]. Regarding cookies, results showed that cookies prepared with fermented apple juice by-products showed the best viability of LAB with values of 1.81 ± 1.13 log CFU/g and 2.42 ± 1.76 log CFU/mL in cookies prepared with barley, and cookies prepared with barley and fortified with *Chlorella vulgais* powder, respectively. Likewise, orange juice by-products exhibited an interesting survival rate of *Lpb. plantarum* strains, with a rate of 2.14 log CFU/mL. It was reported that LAB can survive and grow in fruit matrices; in line with our findings, it was reported that *Lpb. plantarum* CECT9567 grew in orange peel, with a concentration of about 7.9 log CFU/mL [42]. Similarly, Azhar and colleagues found that *Limosilactobacillus fermentum* O1.1 grew rapidly in orange peel medium and banana peels due to the presence of natural carbohydrates, proteins, and fibres [43]. Further studies reported a limited growth of LAB strains in orange-based medium due to the presence of limonene and terpenes, which exhibit antimicrobial activity [43]. The microbiological analysis showed no pathogens in the plant-based beverages.

The antioxidant potential was estimated using DPPH measurement, and results are summarized in the table below (Table 19). Findings revealed that cookies fortified with barley and fermented fruit juice by-products exhibit antioxidant activity ranging from 26.80 ± 1.28 mg Trolox eq./100 g to 33.76 ± 1.04 mg Trolox eq./100 g, with cookies prepared with fermented apple juice matrices exhibiting the highest antioxidant potential. Compared to barley cookies (control), cookies fortified with fermented fruit juice matrices exhibited higher antioxidant activity. Regarding cookies prepared with 3% *Chlorella vulgaris*, all samples showed DPPH activity that was significantly higher than that of all plant-based beverage samples. These findings might be explained by the presence of Chlorella vulgaris, which has been widely highlighted in the literature for its functional and nutritional properties [44]. However, our current results showed no significant difference between cookies with algae powder and those formulated with algae powder and fermented fruit juice by-products. A significant difference was detected (*p* < 0.001) between the plant-based formulation, with only algae (control) and all plant-based samples containing fermented fruit juice matrices.

Orange juice by-products are mostly destined for animal feed or for compound extraction [45]. Recent findings reported higher antioxidant capacity and phenolic content in orange peels and pomace than in the orange fruit. Therefore, orange juice by-products can represent a cost-effective strategy for developing novel functional foods and reducing industry waste. The current paper focuses on the use of orange juice matrices in cookies and plant-based beverage formulations. Indeed, after the lactic fermentation of juice by-products, the fermented juice matrices are introduced as functional ingredients for food fortification. Total antioxidant and phenolic compounds were measured in the final product accordingly. In our study, the total phenolic compounds ranged from 46.43 ± 0.87 mg GAE/100 g to 56.56 ± 0.42 mg GAE/100 g in baked cookies prepared with barley and in cookies with 3% *Chlorella* addition (Table 20). Regarding plant-based beverages, the total phenolic compound content ranged from 48.12 ± 0.42 mg GAE/100 g to 53.61 ± 1.69 mg GAE/100 g, with fermented orange juice by-products exhibiting the highest content. In line with these findings, Caponio and colleagues reported that orange peel flour provided 19.08 ± 0.83 mg GAE/g, while, in muffins enriched with 6% (*w*/*w*) orange peel flour, it accounted for 3.32 ± 0.17 mg GAE/g [46]. In barley cookies, fermented apple juice by-products showed the lowest total phenolic content; indeed, it was reported that reformulated biscuits with a 20% addition of apple matrices contained 36.03 ± 1.85 µg/g [47]. Further study revealed that the addition of algae significantly increased the total phenolic compound content to 6.41 mg GAE/g in fortified cookies [48]. Surprisingly, only cookies prepared with 3% *Chlorella* and fermented tomato juice had a higher phenolic compound content than those prepared with fermented tomato juice alone. In fact, the phenolic compound content in cookies prepared with fermented orange juice was significantly higher than the phenolic content in cookies prepared with 3% *Chlorella* and fermented apple juice by-products. Zooming in on plant-based beverages, total phenolic content ranged from 48.12 ± 0.42 mg GAE/100 g to 53.61 ± 1.69 mg GAE/100 g, and statistical analysis showed no significant difference among the samples.

*Lpb. plantarum* strains isolated from fermented tomato puree showed the highest antioxidant activity at a concentration of 10^9^ CFU/mL and an ability to metabolize a variety of carbohydrates [49]. Likewise, at a dose of 10^10^ CFU/mL of *Lpb. plantarum*, C88 DPPH scavenging and hydroxyl radical activities were counted to 53.05% and 44.31%, respectively [50]. In oat substrate, lactic acid fermentation is widely applied due to its potential to enhance nutritional quality and cost-effectiveness. Recent findings revealed the versatile potential of *Lpb. plantarum* strains to various plant-based substrates [51,52].

### 3.5. Limitations and Future Perspectives

While the developed machine learning models demonstrated reliable predictive capabilities, a primary limitation of the present study is the relatively small size of the experimental dataset used for model training. This constraint stems directly from the significant experimental complexity, substantial resource requirements, and time-intensive nature of the multi-well biological assays necessary to generate precise kinetic data for various strain–matrix combinations. To mitigate the risk of overfitting and ensure robust predictions, rigorous cross-validation and dimensionality reduction techniques were integrated into the computational pipeline. Nevertheless, future research efforts should prioritize scaling up data collection to expand the training dataset. Incorporating a broader range of experimental conditions, such as additional temperature profiles and varied inoculation rates, will be crucial to further enhance the model’s generalizability and predictive accuracy for industrial scale-up applications.

Furthermore, while the model demonstrated high accuracy during the validation trial at 32 °C with an inoculum rate of 0.1, independent validation across a wider array of temperatures and inoculum levels remains necessary to fully confirm the model’s generalizability under diverse operational conditions.

## 4. Conclusions

The current study demonstrates the feasibility of integrating agro-industrial by-products from tomato-, apple-, and orange-processing into a sustainable valorization strategy based on lactic fermentation and data-driven modelling. The application of selected *Lactiplantibacillus plantarum* strains enabled efficient microbial growth across heterogeneous plant matrices, confirming their suitability for the bioprocessing of fruit-derived waste streams, while the development of machine learning-assisted models can be a feasible tool for a predictive framework for describing fermentation kinetics under controlled conditions. Although variability in predictive accuracy was observed across matrices and growth phases, overall error levels remained acceptable (RMSE: 0.143–7.155), supporting the applicability of these models for process optimization.

Overall, this work provides an integrated approach combining microbial biotechnology and machine learning to optimize fermentation processes and support sustainable food innovation. Future studies should focus on optimizing microbial growth modelling and expanding the modelling framework to additional substrates and microbial strains for broader industrial applicability.

## Figures and Tables

**Table 1:** List of the LAB strains used for the present study.

N°	Strain Coding	Strain Name	Origin Source	Accession Number
1	MM1	*Lpb. plantarum* MM1	Wildflower honey	PZ671232
2	MM9	*Lpb. plantarum* MM9	Wildflower honey	PZ671231
3	CECT8944	*Lpb. plantarum* CECT8944	Monastrell wine must, Spain	KC545945
4	SF2A35B	*Lpb. plantarum* SF2A35B	Sour cassava, South America	LMVD00000000.1
5	SD47	*Lpb. plantarum* SD47	Sourdough	PZ671608
6	ATCC8014	*Lpb. plantarum* ATCC 8014	Corn silage	PRJNA247443
7	WCFS1	*Lpb. plantarum* WCFS1	Human saliva	AL935263
8	B01	*Lpb. plantarum* B01	Sourdough Source	PZ671661
9	M5MA1	*Lpb. plantarum* M5MA1	Chicha	OKQP00000000
10	V0102121A9	*Lpb. plantarum* V0102121A 9	Wine	PV163018.1

**Table 2:** Best combination lactic acid bacteria strain and fruit by-product juices.

	Matrix	Selected *Lpb. plantarum* Strain
1	Apple	SD47
2	Tomato	SF2A35B
3	Orange	SD47

**Table 3:** Cookies’ formulation ingredients. Solid ingredients are expressed as g/100 g, and liquid ingredients are expressed as mL/100 g.

Ingredients	Standard Cookies	Cookies + 3% *Chlorella vulgaris* (g/100 g)
Barley	49	46
Algae *Chlorella* powder	0	3
Sugar	20	20
Margarine	20	20
Water	5	5
Fermented fruit juices’ by-products	5	5
Sodium bicarbonate	1	1

**Table 4:** Experimental conditions were conducted on an orange matrix.

Condition	Temperature	Bacterial Inoculum Rate
1	30 °C	0.2 (=2 × 10^8^ CFU/mL)
2	35 °C	0.2 (=2 × 10^8^ CFU/mL)
3	40 °C	0.2 (=2 × 10^8^ CFU/mL)
4	35 °C	1.0 (=10^8^ CFU/mL)
5	30 °C	10.0 (=10^9^ CFU/mL)
6	35 °C	10.0 (=10^9^ CFU/mL)
7	40 °C	10.0 (=10^9^ CFU/mL)

**Table 5:** Selected MaxAvg values for each strain on the orange matrix for each experimental condition tested.

Temperature—Inoculum Rate	ATCC8014	SF2A35B	CECT8944	M5MA1	V0102121A9	B01	SD47	MM9	MM1	WCFS-1
30 °C—0.2	0.454	0.462	0.451	0.467	0.576	0.466	0.258	0.530	0.541	0.775
30 °C—1.0	0.632	0.501	0.589	0.63	0.659	0.5	2.378	0.712	1.625	0.508
35 °C—0.2	0.330	0.317	0.429	0.317	0.375	0.281	0.854	0.636	1.045	0.345
35 °C—1.0	0.655	0.24	0.824	0.953	0.650	0.373	1.14	0.853	1.073	0.722
40 °C—0.2	0.230	0.247	0.225	0.208	0.245	0.224	0.295	0.289	0.307	0.207
40 °C—1.0	1.256	0.642	0.923	0.489	0.543	0.414	1.004	1.481	0.590	0.527
40 °C—10.0	1.492	1.307	1.219	0.312	1.379	0.699	1.525	1.569	1.581	1.165

**Table 6:** Selected vMaxAvg values for each strain on the orange matrix for each experimental condition tested.

Temperature—Inoculum Rate	ATCC8014	SF2A35B	CECT8944	M5MA1	V0102121A9	B01	SD47	MM9	MM1	WCFS-1
30 °C—0.2	0.325	0.209	0.308	0.14	0.263	0.385	0.236	0.253	0.246	0.219
30 °C—1.0	0.263	0.424	0.267	0.731	0.373	0.422	0.05	0.303	0.235	0.417
35 °C—0.2	0.420	1.026	0.234	0.155	0.404	0.12	0.248	0.337	0.238	0.331
35 °C—1.0	0.670	0.391	0.644	0.55	0.504	0.384	0.943	0.601	0.792	0.726
40 °C—0.2	0.299	0.321	0.363	0.358	0.318	0.273	0.324	0.367	0.277	0.261
40 °C—1.0	0.425	0.35	0.557	0.877	0.455	0.771	0.934	0.547	0.713	0.732
40 °C—10.0	0.212	0.21	0.263	0.329	0.199	0.257	0.236	0.239	0.245	0.153

**Table 7:** LagAvg values for each strain on the orange matrix for each experimental condition tested.

Temperature—Inoculum Rate	ATCC8014	SF2A35B	CECT8944	M5MA1	V0102121A9	B01	SD47	MM9	MM1	WCFS-1
30 °C—0.2	9.674	47.988	14.468	56.196	10.224	12.444	20.631	14.244	15.271	30.817
30 °C—1.0	50.875	47.121	50.628	28.248	47.535	47.369	8.107	48.420	68.153	47.272
35 °C—0.2	21.225	36.52	26.065	21.188	16.038	7.093	15.747	15.439	31.982	16.069
35 °C—1.0	36.406	44.536	25.948	47.864	32.397	43.856	8.213	27.142	7.765	25.882
40 °C—0.2	10.645	14.262	8.994	14.121	12.450	18.933	9.148	10.634	13.122	15.398
40 °C—1.0	45.143	59.542	26.692	28.629	36.804	38.356	7.521	39.667	28.214	27.692
40 °C—10.0	8.005	3.346	3.556	11.82	2.462	8.469	10.946	2.226	2.658	1.333

**Table 8:** Predicted and experimental MaxAvg values for all the strains across different fruit matrices at 32 °C and 0.1 inoculum rate (10^8^ CFU/mL).

	Strain Code									
	ATCC8014	SF2A35B	CECT8944	M5MA1	V0102121A9	B01	SD47	MM9	MM1	WCFS-1
	Prediction findings									
Apple	1.123 ± 0.013	1.343 ± 0.013	0.238 ± 0.021	0.642 ± 0.003	0.449 ± 0.030	0.194 ± 0.002	0.605 ± 0.013	0.529 ± 0.010	1.212 ± 0.042	0.334 ± 0.032
Tomato	1.699 ± 0.027	1.486 ± 0.012	1.352 ± 0.009	1.668 ± 0.040	1.675 ± 0.021	1.507 ± 0.040	1.204 ± 0.063	1.666 ± 0.016	1.689 ± 0.034	1.671 ± 0.045
Orange	0.856 ± 0.012	0.463 ± 0.007	0.607 ± 0.013	0.761 ± 0.019	0.841 ± 0.033	0.668 ± 0.053	0.839 ± 0.005	0.632 ± 0.012	0.539 ± 0.063	0.919± 0.048
	Experiment findings									
Apple	1.293 ± 0.012	1.613 ± 0.012	0.402 ± 0.021	0.597 ± 0.01	0.489 ± 0.01	0.212 ± 0.01	0.584 ± 0.01	0.585 ± 0.03	1.895 ± 0.054	0.464 ± 0.012
Tomato	2.362 ± 0.011	1.84	0.962 ± 0.022	1.701 ± 0.01	0.975 ± 0.03	0.941 ± 0.01	1.646 ± 0.01	1.231 ± 0.04	2.483 ± 0.06	0.948 ± 0.054
Orange	0.357 ± 0.001	0.619	0.546 ± 0.01	0.303 ± 0.02	0.63 ± 0.01	0.302 ± 0.01	0.57 ± 0.01	0.353 ± 0.012	0.407 ± 0.04	0.618 ± 0.014

**Table 9:** Predicted and experimental vMaxAvg values for all the strains across different fruit matrices at 32 °C and 0.1 inoculum rate (10^8^ CFU/mL).

	Strain Code									
	ATCC8014	SF2A35B	CECT8944	M5MA1	V0102121A9	B01	SD47	MM9	MM1	WCFS-1
	Prediction findings									
Apple	0.300 ± 0.003	0.41 ± 0.003	0.398 ± 0.004	0.273 ± 0.007	0.320 ± 0.011	0.267 ± 0.018	0.400 ± 0.013	0.420 ± 0.015	0.405 ± 0.022	0.328 ± 0.016
Tomato	0.475 ± 0.029	0.429 ± 0.018	0.550 ± 0.002	0.594 ± 0.005	0.407 ± 0.020	0.565 ± 0.005	0.396 ± 0.006	0.436 ± 0.008	0.641 ± 0.013	0.498 ± 0.004
Orange	0.364 ± 0.009	0.344 ± 0.016	0.217 ± 0.013	0.289 ± 0.007	0.267 ± 0.003	0.293 ± 0.031	0.373 ± 0.032	0.365 ± 0.018	0.428 ± 0.013	0.326 ± 0.006
	Experiment findings									
Apple	0.441 ± 0.003	0.374 ± 0.04	0.379 ± 0.020	0.339 ± 0.021	0.641 ± 0.011	0.412 ± 0.007	0.408 ± 0.015	0.253	0.56 ± 0.002	0.27
Tomato	0.673 ± 0.01	1.198 ± 0.007	0.299 ± 0.015	0.533 ± 0.011	0.60 ± 0.013	0.293 ± 0.014	0.162 ± 0.004	0.367	0.40 ± 0.01	0.567
Orange	0.085 ± 0.009	0.16 ± 0.004	0.267 ± 0.011	1.472 ± 0.004	0.227 ± 0.031	0.393 ± 0.010	0.177 ± 0.014	0.094 ± 0.001	0.62 ± 0.022	0.334

**Table 10:** Predicted and experimental LagAvg values for all the strains across different fruit matrices at 32 °C and 0.1 inoculum rate (10^8^ CFU/mL).

	Strain Code									
	ATCC8014	SF2A35B	CECT8944	M5MA1	V0102121A9	B01	SD47	MM9	MM1	WCFS-1
	Predicted findings									
Apple	14.294 ± 1.003	12.12 ± 2.122	14.697 ± 1.494	13.718 ± 1.206	12.155 ± 1.294	18.382 ± 2.463	11.456 ± 2.324	17.14 ± 0.906	17.141 ± 1.045	14.865 ± 1.463
Tomato	6.453 ± 0.373	6.954 ± 0.749	6.142 ± 2.193	4.708 ± 0.203	6.952 ± 0.685	6.315 ± 0.243	6.213 ± 0.533	6.865 ± 1.353	5.529 ± 1.303	6.849 ± 1.036
Orange	8.209 ± 0.859	9.3325 ± 1.849	8.725 ± 0.365	10.391 ± 1.376	8.586 ± 1.993	9.097 ± 0.343	12.504 ± 0.321	12.727 ± 1.465	13.731 ± 1.003	7.832 ± 0.345
	Experiment findings									
Apple	14.525 ± 2.005	12.873 ± 0.012	16.91 ± 0.021	14.51 ± 0.037	15.835 ± 0.021	12.859 ± 0.01	12.952 ± 0.024	11.37 ± 0.10	14.761 ± 0.301	15.383
Tomato	3.729 ± 0.021	3.808 ± 0.022	8.665 ± 0.022	5.002 ± 0.014	8.26 ± 0.041	9.436 ± 0.02	1.615 ± 0.034	5.576 ± 0.314	3.699 ± 0.014	7.96
Orange	12.36 ± 0.021	9.6 ± 0.03	15.993 ± 0.061	15.365 ± 0.017	13.03 ± 0.001	17.164 ± 0.03	12.723 ± 0.07	2.99 ± 0.201	2.956 ± 0.347	16.281

**Table 11:** Prediction and experimental findings of viable bacterial counts for the selected fermentation combinations. Values are expressed in log (CFU/mL). Confidence levels of the prediction findings are mentioned in the next table (Table 12).

	Strain Code									
	MM1	MM9	CECT9847	SF2A	SD47	ATCC8014	WCF1	B01	M5M1	V010212
	Prediction findings—microbial growth (log (CFU/mL))									
Apple	10.12 ± 0.206	5.29 ± 0.013	4.98 ± 0.04	11.12 ± 0.123	8.85 ± 0.304	10.77 ± 0.064	7.77 ± 0.01	9.12 ± 0.024	6.42 ± 0.01	6.49 ± 0.022
Tomato	6.44 ± 0.102	6.78 ± 0.041	13.52 ± 0.104	6.95 ± 0.214	12.04 ± 0.002	6.85 ± 0.013	6.88 ± 0.031	6.32 ± 0.041	5.94 ± 0.031	6.98 ± 0.011
Orange	5.39 ± 0.108	6.38 ± 0.04	6.10 ± 0.132	7.22 ± 0.130	8.39 ± 0.025	8.56 ± 0.021	6.20 ± 0.012	6.70 ± 0.014	7.77 ± 0.03	8.44 ± 0.052
	Experiment findings—microbial growth (log (CFU/mL))									
Apple	9.30 ± 8.75	8.79 ± 7.58	8.63 ± 7.6	9.22 ± 8.72	**8.78 ± 7.99**	8.71 ± 7.09	8.72 ± 7.22	8.41 ± 7.45	8.68 ± 48	8.72 ± 7.21
Tomato	9.40 ± 8.45	9.11 ± 8.67	9.00 ± 8.62	**8.94 ± 7.52**	9.20 ± 8.43	9.37 ± 8.64	8.98 ± 8.06	8.99 ± 8.21	9.29 ± 7.94	8.99 ± 7.05
Orange	8.65 ± 7.22	8.54 ± 7.4	8.73 ± 8.10	8.79 ± 6.65	**8.76 ± 7.1**	8.54 ± 6.04	8.79 ± 7.81	8.49 ± 7.20	8.51 ± 6.5	8.80 ± 6.9

**Table 12:** Confidence levels of prediction findings and experiment results for LAB strains across different fruit matrices at 32 °C with 0.1 bacterial inoculum rate (10^8^ CFU/mL).

	Strain Code									
	ATCC8014	SF2A35B	CECT8944	M5MA1	V0102121A9	B01	SD47	MM9	MM1	WCFS-1
	Prediction findings									
Apple	0.569	0.672	0.704	0.675	0.865	0.612	0.894	0.753	0.62	0.503
Tomato	0.562	0.511	0.415	0.635	0.643	0.488	0.367	0.471	0.532	0.665
Orange	0.317	0.367	0.415	0.439	0.367	0.314	0.437	0.348	0.319	0.367
	Experiment findings									
Apple	0.996	0.995	0.824	0.939	0.845	0.606	0.967	0.936	0.982	0.923
Tomato	0.921	0.911	0.992	0.932	0.998	0.994	0.905	0.988	0.956	0.998
Orange	0.78	0.974	0.807	0.657	0.984	0.989	0.91	0.68	0.703	0.941

**Table 13:** RMSE of the three parameters between the experimental and predicted findings.

Parameter/Matrix	Apple	Tomato	Orange
MaxAvg	0.249	0.806	0.304
vMaxAvg	0.143	0.377	0.450
LagAvg	2.995	3.066	7.155

**Table 14:** MaxAvg predictions for the ML models for each strain on the tomato matrix in the experimental conditions tested in the laboratory.

Temperature—Inoculum Rate	ATCC8014	SF2A35B	CECT8944	M5MA1	V0102121A9	B01	SD47	MM9	MM1	WCFS-1
30 °C—0.1	1.699	1.486	1.351	1.668	1.675	1.507	1.204	1.666	1.689	1.671
30 °C—0.2	1.699	1.438	1.727	1.724	1.675	1.669	1.624	1.810	1.839	1.997
30 °C—1.0	2.014	1.927	2.234	2.305	1.901	2.063	1.789	2.141	2.172	2.204
35 °C—0.2	1.813	1.588	1.797	1.798	1.761	1.799	1.721	1.801	1.773	1.761
35 °C—1.0	1.919	1.992	1.734	2.058	1.687	1.779	1.782	1.807	2.066	1.800
37 °C—0.1	1.813	1.209	1.437	1.529	1.761	1.612	1.325	1.881	1.723	1.716
40 °C—0.2	1.763	1.288	1.606	1.668	1.501	1.678	1.635	1.698	1.842	1.393
40 °C—1.0	1.913	2.143	1.627	1.820	1.701	1.727	1.665	1.741	2.120	1.720
40 °C—10.0	1.675	1.579	1.682	1.742	1.815	1.794	2.036	1.728	2.120	1.905

**Table 15:** vMaxAvg predictions for the ML models for each strain on tomato matrix in the experimental conditions tested in laboratory.

Temperature—Inoculum Rate	ATCC8014	SF2A35B	CECT8944	M5MA1	V0102121A9	B01	SD47	MM9	MM1	WCFS-1
30 °C—0.1	0.475	0.429	0.549	0.594	0.408	0.565	0.396	0.436	0.641	0.498
30 °C—0.2	0.474	0.346	0.548	0.601	0.407	0.418	0.469	0.299	0.245	0.265
30 °C—1.0	0.342	0.342	0.348	0.349	0.339	0.343	0.347	0.344	0.346	0.349
35 °C—0.2	0.545	0.398	0.770	0.644	0.585	0.435	0.485	0.501	0.734	0.594
35 °C—1.0	0.408	0.402	0.414	0.406	0.473	0.397	0.376	0.398	0.536	0.397
37 °C—0.1	0.545	0.342	0.598	0.460	0.585	0.525	0.419	0.551	0.692	0.601
40 °C—0.2	0.430	0.327	0.491	0.548	0.532	0.247	0.416	0.477	0.727	0.375
40 °C—1.0	0.408	0.373	0.410	0.458	0.591	0.277	0.366	0.418	0.525	0.379
40 °C—10.0	0.358	0.360	0.295	0.361	0.359	0.361	0.301	0.346	0.525	0.356

**Table 16:** LagAvg predictions for the ML models for each strain on tomato matrix in the experimental conditions tested in laboratory.

Temperature—Inoculum Rate	ATCC8014	SF2A35B	CECT8944	M5MA1	V0102121A9	B01	SD47	MM9	MM1	WCFS-1
30 °C—0.1	6.453	6.954	6.142	4.708	6.977	6.315	6.213	6.865	5.528	6.849
30 °C—0.2	6.453	7.996	7.078	5.416	6.977	7.293	7.514	12.001	15.989	22.392
30 °C—1.0	8.533	8.553	7.585	7.260	8.929	8.235	13.035	8.071	7.643	7.384
35 °C—0.2	4.906	7.496	6.079	5.235	5.501	7.443	5.039	4.820	5.068	6.710
35 °C—1.0	8.209	9.382	8.190	8.261	6.769	10.010	9.204	10.234	11.945	10.338
37 °C—0.1	4.906	10.542	6.069	6.166	5.501	7.589	5.784	5.220	6.034	8.231
40 °C—0.2	6.144	7.506	5.849	6.208	6.741	7.635	5.916	6.166	7.148	8.029
40 °C—1.0	9.399	7.799	8.897	10.633	6.809	11.305	10.028	10.759	3.777	9.521
40 °C—10.0	4.451	3.719	10.348	4.859	4.310	4.189	4.456	5.199	3.773	3.612

**Table 17:** pH measurement after plant-based beverage fermentation.

Combination Strain–Juice	pH T0	pH T1
Apple-*Lpb. plantarum* SD47	6.00	3.8
Tomato-*Lpb. plantarum* SF2A35B	6.50	3.75
Orange-*Lpb. plantarum* SD47	6.33	3.75
Barley (indigenous microbiota)	6.00	6.00

**Table 18:** Viable cell concentration in food matrices. CFU counts of viable microbes of each combination in the corresponding microbiological medium of growth. Colony counting is expressed as log CFU/mL for barley beverage and as log CFU/g for cookies. PB: Plant-based beverage; CB: cookies formulated with barley; CBA: cookies prepared with barley and algae; N.D: Not detected. * *p* < 0.0001 vs. PB Apple-SD47 PCA and PB Apple-SD47 PDA counts; ^A^ *p* < 0.0001 vs. PB Orange-SD47 MRS and PB Orange-SD47 PDA counts; ^B^ *p* < 0.0001 vs. PB Orange-SD47 PCA and PB Orange-SD47 PDA counts; ^C^ *p* < 0.0001 vs. PB Tomato-SF2A35B MRS and PB Tomato-SF2A35B PDA counts; ^D^ *p* < 0.05 vs. CB Apple-SD47 MRS, CB Orange-SD47 MRS, and CB Tomato-SF2A35B MRS counts; ^E^ *p* < 0.05 vs. CB Orange-SD47 PDA count; ^F^ *p* < 0.05 vs. CBA Apple-SD47 PCA and CBA Tomato-SF2A35B PCA counts; ^G^ *p* < 0.05 vs. CBA Apple-SD47 MRS and Orange-SD47 MRS counts; ^H^ *p* < 0.05 vs. PB Apple-SD PDA count, PB Orange-SD47 PDA, and PB Tomato-SF2A35B PDA count. No significant difference in bacterial counts (MRS, PCA, and PDA) between cookies prepared with barley (CB) and cookies prepared with barley and fortified with *Chlorella* powder (CBA).

Combination (Fruit Matrix–Strain Code)	Food	PCA	MRS	PDA	SMAC	PALCAM
		Total Bacterial Count	LAB	Moulds and Yeasts	Pathogenic *E. coli*	*Listeria* spp.
Apple-SD47	PB	3.15 ± 2.37	10.91 ± 10.37 *	N.D	N.D	N.D
Orange-SD47	PB	4.23 ± 2.84 ^A^	10.71 ± 9.74 ^B^	N.D	N.D	N.D
Tomato-SF2A35B	PB	7.85 ± 5.94 ^C^	11.47 ± 9.70	0.8 ± 0.09	N.D	N.D
Barley (indigenous microbiota)	PB	N.D	N.D	6.35 ± 2.48 ^H^	N.D	N.D
Barley (indigenous microbiota) + Algae 1.5%	PB	N.D	N.D	4.35 ± 3.89	N.D	N.D
Apple-SD47	CB	1.2 ± 0.57	1.81 ± 1.13	0.63 ± 0.38	N.D	N.D
Orange-SD47	CB	0.74 ± 0.2	1.75 ± 1.12	1.46 ± 0.91	N.D	N.D
Tomato-SF2A35B	CB	0.97 ± 0.32	1.48 ± 1.1	1.12 ± 0.7	N.D	N.D
Barley (indigenous microbiota)	CB	0.0 ± 0.0	0.0 ± 0.0 ^D^	0.0 ± 0.0 ^E^	N.D	N.D
Apple-SD47	CBA	2.15 ± 1.74	2.42 ± 1.76	0.97 ± 0.21	N.D	N.D
Orange-SD47	CBA	1.62 ± 1.4	2.14 ± 1.09	0.21 ± 0.02	N.D	N.D
Tomato-SF2A35B	CBA	1.98 ± 057	0.95 ± 0.52	0.42 ± 0.10	N.D	N.D
Barley (indigenous microbiota)	CBA	N.D	N.D	N.D	N.D	N.D
Barley (indigenous microbiota) + Algae 3%	CBA	0.0 ± 0.0 ^F^	0.0 ± 0.0 ^G^	N.D	N.D	N.D

**Table 19:** Antioxidant activity of food products.

Antioxidant Content in Food Product (mg Trolox eq./100 g)		
Food Product	DPPH	Combination
Cookies Barley	33.76 ± 1.04 *^,a^	Apple-SD47
Cookies Barley	26.80 ± 2.11	Orange-SD47
Cookies Barley	26.80 ± 1.28	Tomato-SF2A35B
Cookies Barley Algae	30.71 ± 0.0 ^b^	Apple-SD47
Cookies Barley Algae	32.38 ± 0.84 ^c^	Orange-SD47
Cookies Barley Algae	32.66 ± 5.45 ^d^	Tomato-SF2A35B
Plant-Based Beverage	23.17 ± 0.84	Apple-SD47
Plant-Based Beverage	24.68 ± 1.67	Orange-SD47
Plant-Based Beverage	23.29 ± 0.48	Tomato-SF2A35B
Cookies Barley (control: barley)	25.13 ± 0.48	Barley
Cookies Barley Algae (control: barley + 3% algae)	39.08 ± 0.84	Barley + 3% Algae
Cookies Barley Algae (control: barley + 3% water)	29.31 ± 1.28	Barley + Aqua
Plant-Based Beverage (control: barley)	20.11 ± 0.48	Barley
Plant-Based Beverage (control: barley + algae)	17.28 ± 0.07	Barley + Algae

* *p* < 0.001 vs. cookies barley orange-SD47 and cookies barley tomato-SF2A35B; ^a^ *p* < 0.0001 vs. plant-based beverage orange-SD47 and plat-based beverage tomato-SF2A35B; ^b^ *p* < 0.05 vs. plant-based beverage apple-SD47 and plant-based beverage orange-SD47 and plant-based beverage tomato- SF2A35B; ^c^ *p* < 0.001 vs. plant-based beverage apple-SD47 and plant-based beverage orange-SD47 and plant-based beverage tomato- SF2A35B; ^d^ *p* < 0.001 vs. plant-based beverage apple-SD47 and plant-based beverage orange-SD47 and plant-based beverage tomato- SF2A35B.

**Table 20:** Total phenolic compound content in food products.

	Total Phenolic Compounds (mg GAE/100 g)	
Cookies Barley	46.43 ± 0.87 ^a^	Apple-SD47
Cookies Barley	56.56 ± 0.42 *	Orange-SD47
Cookies Barley	53.33 ± 3.81	Tomato-SF2A35B
Cookies Barley Algae	45.73 ± 0.49 ^b^	Apple-SD47
Cookies Barley Algae	56.14 ± 0.84	Orange-SD47
Cookies Barley Algae	55.86 ± 2.97	Tomato-SF2A35B
Plant-Based Beverage	48.12 ± 0.42	Apple-SD47
Plant-Based Beverage	53.61 ± 1.69	Orange-SD47
Plant-Based Beverage	53.19 ± 2.96	Tomato-SF2A35B

* *p* < 0.001 vs. cookies barley algae Apple-SD47; ^a^ *p* < 0.05 vs. plant-based beverage apple-SD47; ^b^ *p* < 0.05 vs. cookies barley algae orange-SD47 and cookies barley algae tomato-SF2A35B.

## Data Availability

The data generated for this study are available on request to the corresponding authors.

## Supplementary Materials

The following supporting information can be downloaded at: https://www.mdpi.com/article/10.3390/foods15152765/s1, Figure S1: the user interface was selected for the decision support system; Figure S2: the user interface prediction results.
