# Supplementary material for: An Integrated Case Study on Fruit By-Products/Waste Management: Lactic Acid Bacteria Development, Artificial Intelligence Smart Implementation, and Reintegration into Food Chain
Source: Foods. 2026 Aug 6;15(15):2765. doi: 10.3390/foods15152765 (PMC13465421; doi:10.3390/foods15152765)
Supplement: Supplementary file 1 [file foods-15-02765-s001.zip › foods-4403310-supplementary.pdf]

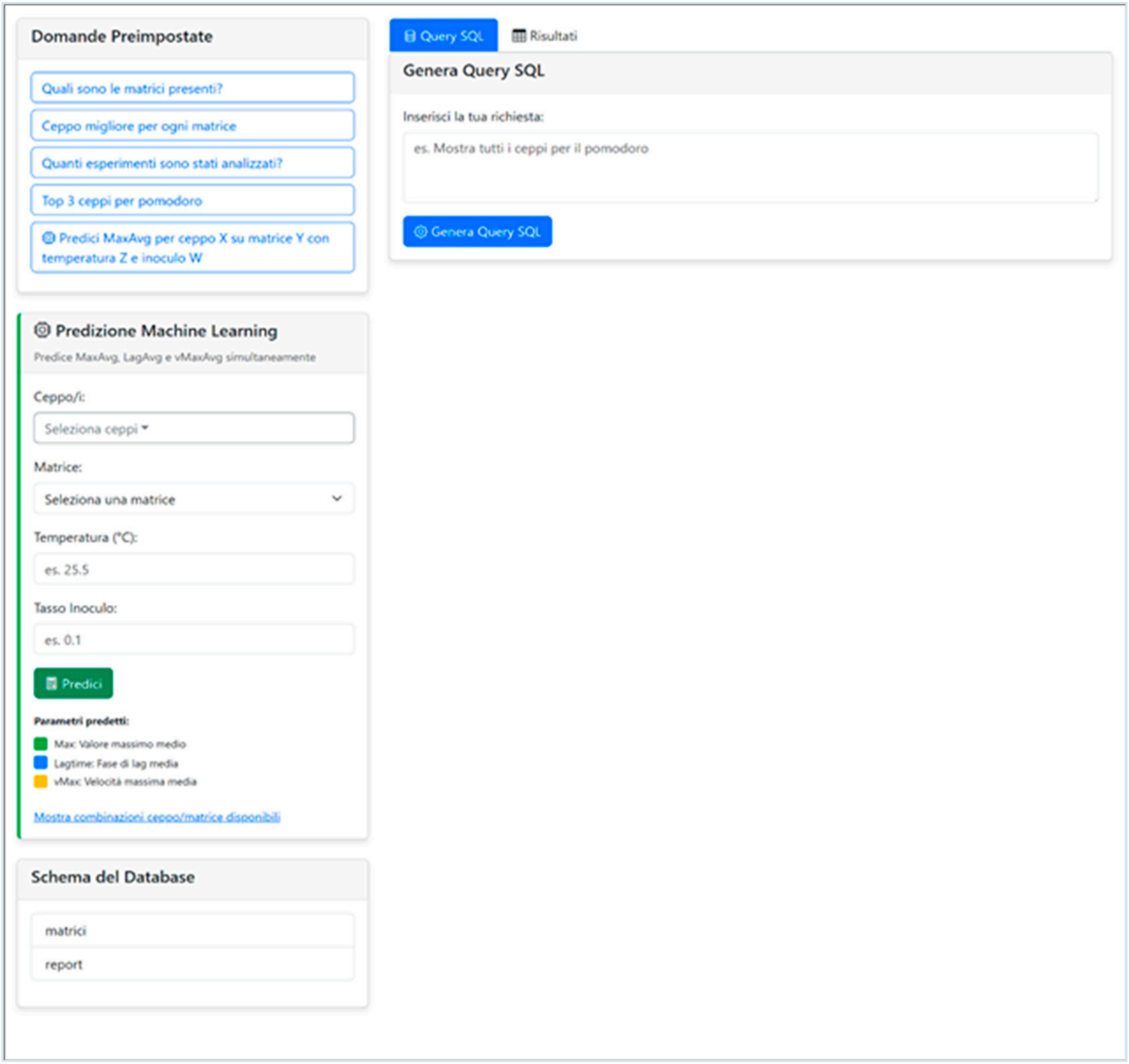

Figure S1. The user interface was selected for the decision support system. Note: The graphical user interface (GUI) of the prototype is presented in its original Italian version, reflecting its initial development and deployment phase tailored for local industrial end-users. The underlying database schemas, natural language processing, and predictive machine learning algorithms operate entirely independently of the front-end language.

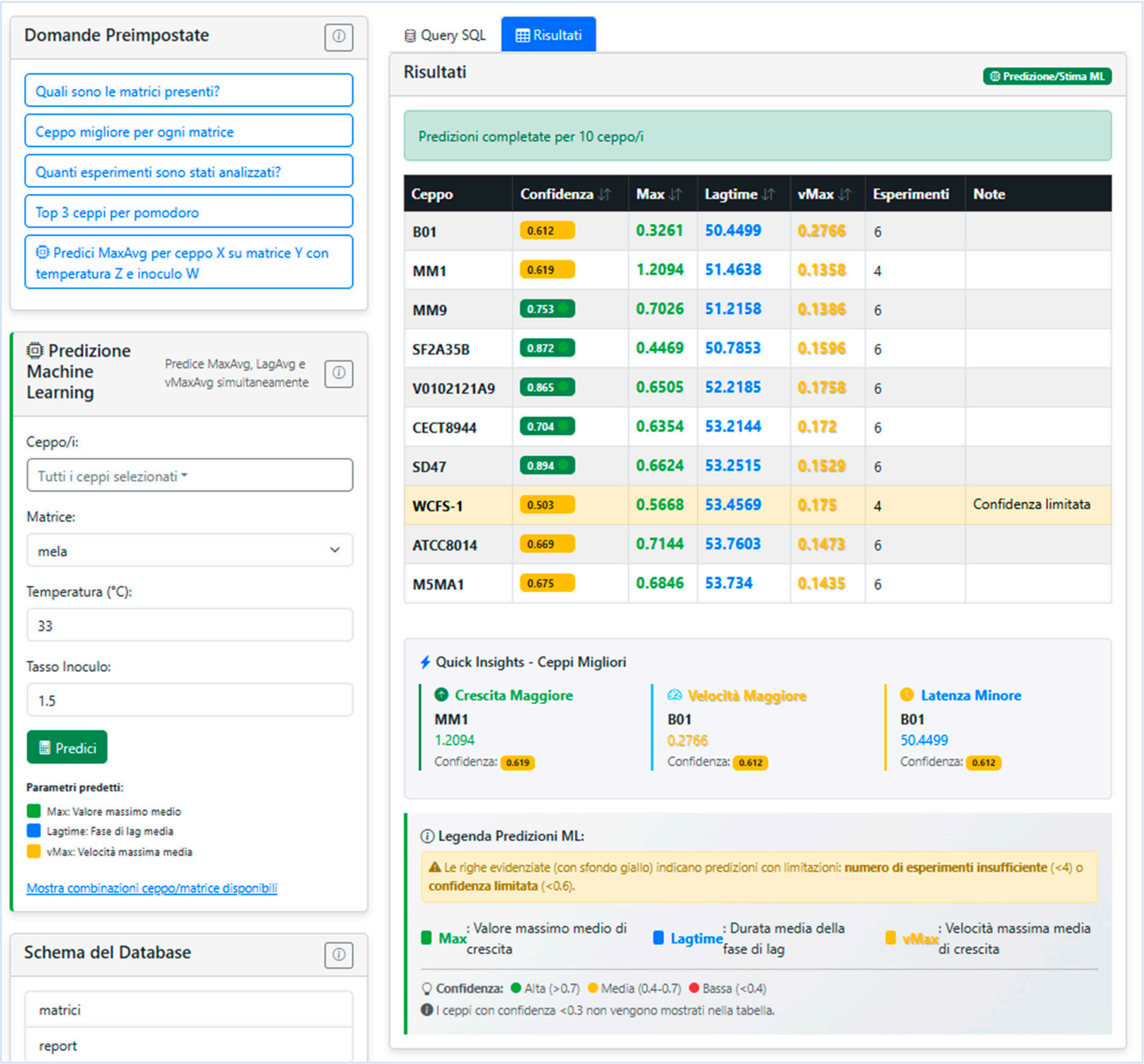

Figure S2. The user interface prediction results.
